# Structural insight into the cooperation of chloroplast chaperonin subunits

**DOI:** 10.1186/s12915-016-0251-8

**Published:** 2016-04-12

**Authors:** Shijia Zhang, Huan Zhou, Feng Yu, Cuicui Bai, Qian Zhao, Jianhua He, Cuimin Liu

**Affiliations:** State Key Laboratory of Plant Cell and Chromosome Engineering, Institute of Genetics and Developmental Biology, Chinese Academy of Sciences, Beijing, 100101 China; Shanghai Institute of Applied Physics, Chinese Academy of Sciences, Shanghai, 201204 China; University of Chinese Academy of Sciences, Beijing, 100101 China

**Keywords:** Chaperonin, Photosynthesis, Protein disassembly, Protein folding, Rubisco

## Abstract

**Background:**

Chloroplast chaperonin, consisting of multiple subunits, mediates folding of the highly abundant protein Rubisco with the assistance of co-chaperonins. ATP hydrolysis drives the chaperonin allosteric cycle to assist substrate folding and promotes disassembly of chloroplast chaperonin. The ways in which the subunits cooperate during this cycle remain unclear.

**Results:**

Here, we report the first crystal structure of *Chlamydomonas* chloroplast chaperonin homo-oligomer (CPN60β1) at 3.8 Å, which shares structural topology with typical type I chaperonins but with looser compaction, and possesses a larger central cavity, less contact sites and an enlarged ATP binding pocket compared to GroEL. The overall structure of Cpn60 resembles the GroEL allosteric intermediate state. Moreover, two amino acid (aa) residues (G153, G154) conserved among Cpn60s are involved in ATPase activity regulated by co-chaperonins. Domain swapping analysis revealed that the monomeric state of CPN60α is controlled by its equatorial domain. Furthermore, the C-terminal segment (aa 484–547) of CPN60β influenced oligomer disassembly and allosteric rearrangement driven by ATP hydrolysis. The entire equatorial domain and at least one part of the intermediate domain from CPN60α are indispensable for functional cooperation with CPN60β1, and this functional cooperation is strictly dependent on a conserved aa residue (E461) in the CPN60α subunit.

**Conclusions:**

The first crystal structure of *Chlamydomonas* chloroplast chaperonin homo-oligomer (CPN60β1) is reported. The equatorial domain maintained the monomeric state of CPN60α and the C-terminus of CPN60β affected oligomer disassembly driven by ATP. The cooperative roles of CPN60 subunits were also established.

**Electronic supplementary material:**

The online version of this article (doi:10.1186/s12915-016-0251-8) contains supplementary material, which is available to authorized users.

## Background

Cellular protein homeostasis is regulated by molecular chaperones [1, 2]. Chaperonins are a subfamily of chaperones which have diverged into two distinct groups [1, 3, 4]. Group I chaperonins, which are found in prokaryotes and in organelles of prokaryotic origin, include GroEL/ES in *E. coli*, Hsp60/10 in mitochondria, and Cpn60/20 in chloroplasts [5–7]. Group II chaperonins are further divided into archaeal types (thermosome) and eukaryotic types (TRiC) [8, 9]. Both chaperonin groups share a similar molecular architecture consisting of two back-to-back stacked rings, with a central cavity in each ring that allows substrates to fold. The structure of group I chaperonin GroEL/ES is well established [10–12] and the mitochondrial Hsp60/10 crystal structure has been recently solved [13]. Both chaperonins are cylindrical structures consisting of 14 identical subunits assembled into two heptameric rings. The crystal structures of GroEL in its various forms (GroEL apo, GroEL/ADP, GroEL/ES/ADP), GroEL mutants, and mini-chaperones have facilitated the understanding of its functional mechanism. Even the structure of mini-chaperones, which were solved in high resolution, provided details of side chains which are involved in the interaction or regulation with other proteins or molecules [10, 11, 14–16]. Each of the 14 individual subunits of the chaperonins has three domains: the apical, the equatorial, and the intermediate hinge domains. In GroEL, the apical domain (amino acid (aa) residues 189–377) recognizes substrate proteins via hydrophobic residues exposed toward the central cavity and interacts with its co-chaperonin GroES [17, 18]. The equatorial domain (aa residues 2–136 and 410–525) contains the ATP binding site and contributes to inter-ring contacts [19]. These two domains are connected by the intermediate hinge domain (aa residues 137–188 and 378–409), which allows rigid body movement.

In the past 20 years, the asymmetric mechanism of GroEL-assisted folding has been supported, i.e. GroES binds to one GroEL ring (Bullet) and the two rings function sequentially [20, 21]. However, the observation of a symmetric GroEL-GroES2 complex, in which both GroEL rings are capped with GroES (Football), leads to the suggestions that the symmetric complexes represent a folding intermediate and that the two GroEL rings fold polypeptides simultaneously [22–26]. Recently, Haldar et al. [27] found that the presence of symmetric GroEL-GroES2 complexes was largely dependent on the fluoro-fluorescence pair used to label the chaperonin system and non-foldable substrates. Whether a sequential or simultaneous folding mechanism is undertaken by chaperonin in vivo is still under investigation.

Chaperonins utilize ATP to drive a conformational cycle that allows them to capture, encapsulate, fold, and release substrate proteins [28, 29]. Binding and hydrolysis of ATP induce both positive (intra-ring) and negative cooperative (inter-ring) actions of GroEL [30]. ATP hydrolysis also promotes the partial disassembly of chloroplast chaperonin into the monomer [31, 32], a phenomenon specific to chloroplast chaperonin. The chloroplast chaperonin was first identified as a Rubisco ‘large subunit binding protein’ [33, 34]. Unlike homo-oligomeric GroEL and mitochondrial Hsp60, chloroplast chaperonin consists of multiple subunits, diverging into two distinct but related α and β types [6, 35]. Cpn60α does not assemble into tetradecameric oligomers unless incorporated into oligomers of Cpn60β subunits [6, 31]. Though the authentic chloroplast chaperonin consists of both subunit types in vivo, homo-oligomeric Cpn60β is functional in refolding model substrates with assistance from co-chaperonin in vitro [6, 36, 37]. Two highly conserved CPN60β oligomers exhibit significantly different biochemical properties [38, 39], e.g. homo-oligomers of CPN60β2, but not CPN60β1, from *Chlamydomonas* completely disassemble into monomers upon ATP hydrolysis. Although new information about chloroplast chaperonin is steadily being published, its structure and complicated regulatory mechanism remain unknown.

Here, we report the first crystal structure of chloroplast homo-oligomer CPN60β1 in its apo form, with a larger molecular diameter than GroEL and novel ATP binding pockets. Domain swapping between CPN60α and CPN60β revealed that the equatorial domain mediates oligomer formation and the two subunits are highly cooperative in forming functional oligomers.

## Results

### Homo-oligomeric CPN60β1 bound to co-chaperonins but could not assist folding of model substrate RrRubisco

**Fig. 1:**
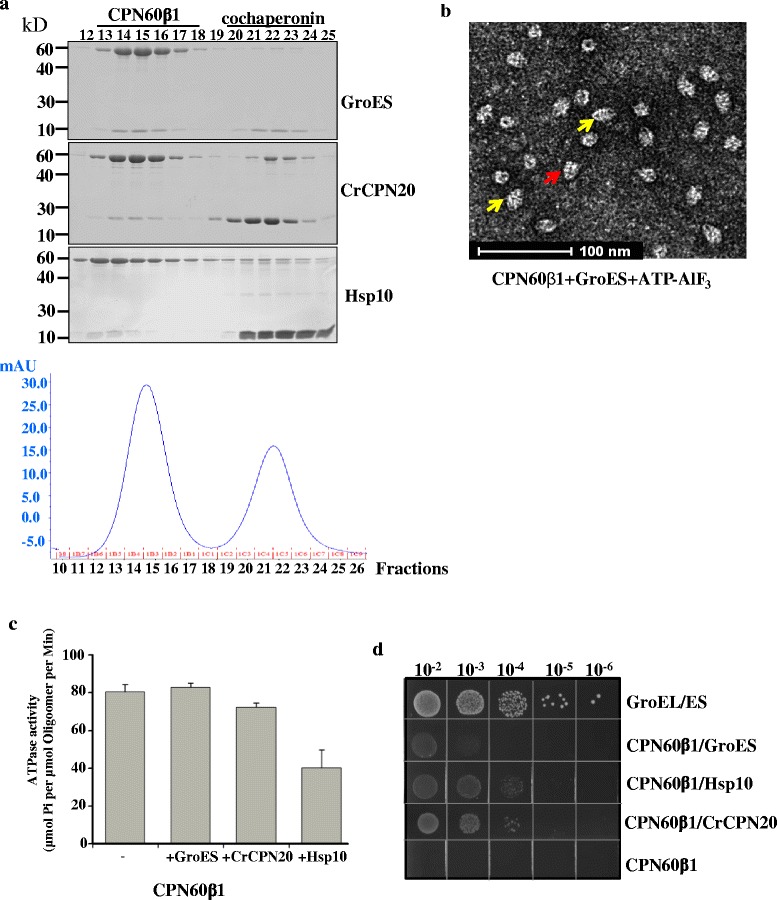
Interaction of homo-oligomeric CPN60β1 with co-chaperonin. **a** Analytical gel filtration of CPN60β1-cochaperonin complexes. A 50-μL reaction mixture containing 2 μM CPN60β1 and 10 μM co-chaperonins, as indicated, was run on a Superdex 200 column with buffer containing 50 μM ADP-AlF_3_. Collected fractions were resolved by SDS-PAGE and stained with Coomassie. A representative picture from the UV trace of gel filtration is shown in the bottom panel. **b** Electron micrograph of CPN60β1 with GroES in the presence of ATP-AlF_3_. The ‘football’ and ‘bullet’ structures are indicated by yellow and red arrows, respectively. **c** ATPase activity of CPN60β oligomers. The ATP hydrolysis rate of 0.2 μM chaperonin in the presence or absence of 0.4 μM co-chaperonins was measured for 10 min at 25 °C. The data was obtained with three independent experimental replicates and standard deviations are shown. **d** Functional complementation of GroEL by CPN60β1. The expression of CPN60β and selected co-chaperonin was induced with 1 mM IPTG in GroEL-deficient *E. coli* strain, MGM100. The strains were grown on medium supplemented with glucose and IPTG at 37 °C for 15 hours

In *Chlamydomonas*, functional CPN60 is composed of three subunits [39]. The individual subunits of CPN60 from *Chlamydomonas* do not complement GroEL function in *E. coli* [39], but it was reported that homo-oligomeric Cpn60β is functional in refolding model substrates in vitro [6, 36]. To investigate if homo-oligomeric CPN60β1 from *Chlamydomonas* is functional in vitro, we first assessed the interaction between CPN60β1 and co-chaperonins by gel filtration. In addition to Cpn10, which is similar to GroES in size, there is a co-chaperonin, Cpn20, which is double the size of GroES in chloroplasts [40, 41]. Both GroES and CPN20 from *Chlamydomonas* (CrCPN20) could in fact form complexes with homo-oligomeric CPN60β1 (Fig. 1a). Furthermore, both ‘bullet’ and ‘football’ structures were observed by electron microscopy after incubation of CPN60β1 with GroES in the presence of ATP-AlF_3_, with ‘football’ structures being prevalent (Fig. 1b) [22, 24]. These results indicate that CPN60β1 interacted with co-chaperonins. GroES and CrCPN20 did not inhibit the ATPase activity of CPN60β1 effectively (Fig. 1c). By contrast, the mitochondrial co-chaperonin Hsp10 from mouse inhibited ATPase activity of CPN60β1 by 50 %, which was consistent with effects observed on PsCpn60β from *Pisum sativum* [37]. Unexpectedly, Hsp10 could not assist homo-oligomeric CPN60β1 in refolding the model substrate RrRubisco (Additional file 1: Figure S1), which is contrary to experimental results obtained using PsCpn60β [37]. In *E. coli*, the cooperation of CPN60β1/Hsp10 could partially complement GroEL/ES function (Fig. 1d). The observations that co-chaperonins bind to homo-oligomeric CPN60β1 and that mitochondrial Hsp10 partially assists CPN60β1 in its chaperonin function suggest that the structure of homo-oligomeric CPN60β1 resembles that of functional hetero-oligomers.

### Crystal structure of the CPN60β1 oligomers

**Fig. 2:**
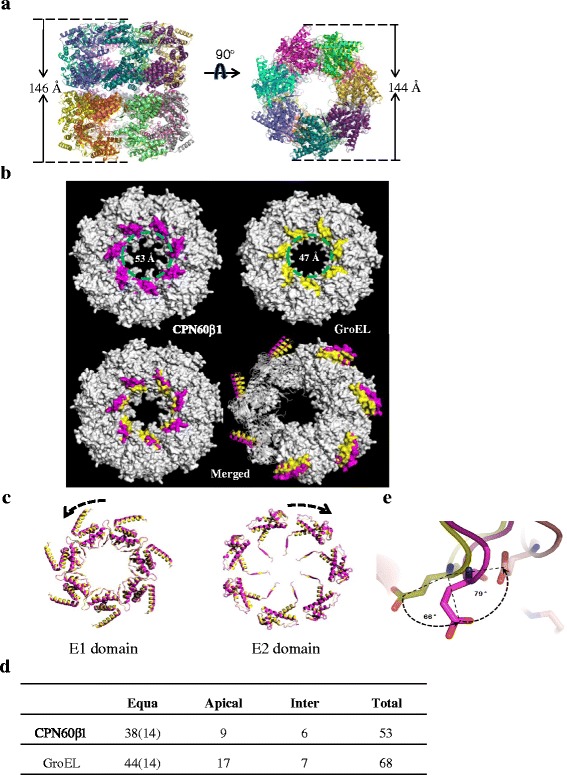
X-ray crystal structures of CPN60β1 oligomers. **a** The side view of the crystal structure perpendicular to the axis of the oligomeric cylinder (*left*) and top view parallel to the axis of the oligomeric cylinder (*right*). Individual subunits are designated by different colors. Estimated structural dimensions are indicated. **b** Comparison of the structures of CPN60β1 oligomer versus GroEL. Position of helix H to helix I is colored in pink in CPN60β1 and yellow in GroEL (*upper* images of each chaperonin alone and *lower left* merged image of both chaperonins) to show the size of the central cavity. The diameter of the central cavities is indicated in the top images. The position of helix K to helix L is highlighted with pink in CPN60β1 and yellow in GroEL (*lower right* merged image of both chaperonins). **c** The bottom view of E1 and E2 domains from the upper single ring. CPN60β1 is colored in pink and GroEL in yellow. **d** Numbers of amino acids involved in the inter- and intra-ring contacts within one subunit. A contact was defined as an atomic distance between two amino acids of less than 4 Å. The number of amino acids in each domain is shown; 14 amino acids (shown in parentheses) in the equatorial domain are involved in inter-ring contacts. **e** Position of E209 in different conformations. The GroEL apo state, the CPN60β1 apo state, and GroEL-ADP state are colored in yellow, pink, and orange, respectively

The crystallization of functional hetero-oligomers of CPN60 did not succeed, whereas the crystallization of CPN60β1 homo-oligomers was achieved by the sandwich method [42]. The crystals belong to space group P21, with 14 subunits per asymmetric unit. The crystal structure of CPN60β1 was solved by molecular replacement with apo GroEL (PDB: 1XCK) [43] as a search model. The electron density map was well fitted in the equatorial domain, but some segments in the apical domain were not visible. We fitted the apical domain structure of CPN60β1 (PDB: 5CDK) to refine the structure. The structural refinement details are listed in Additional file 1: Table S1. In overall architecture, CPN60β1 presents as a typical type I chaperonin, with a 7-fold symmetrical cylinder structure consisting of two stacked rings composed of seven subunits (Fig. 2a). Similar to GroEL subunits, each CPN60 subunit consists of an equatorial, intermediate, and apical domain. However, there are three features that distinguish the chloroplast chaperonin: (1) The central cavity of CPN60 is relatively large; analysis of helices H-I (aa residues 231–273, highlighted in Fig. 2b), which reside on the inner surface of the cavity and are involved in both binding polypeptides and interaction with co-chaperonin [11, 15, 18], revealed that the diameter of the central cavity of CPN60β1 oligomer is 6 Å larger than that of GroEL (Fig. 2b). (2) The structure of CPN60β1 is loosely compacted; a closer view revealed that the equatorial domain of CPN60β1 extends away from the central axis, with E1 (aa residues 2–136) and E2 (aa residues 410–525) domains rotating counterclockwise and clockwise, respectively, in comparison to the equatorial domain of GroEL, although the overall structures merged well (Fig. 2c). These observations indicate a looser compaction of CPN60β1 relative to GroEL. The inter-subunit interface area in the same ring was calculated by the PISA program [44]. The area between two subunits in CPN60β1 is 1,428 Å^2^, less than 10 % that in GroEL (1,573 Å^2^; 1XCK). Furthermore, we counted the amino acids involved in inter-subunit contacts carefully by setting the distance of atoms from different chains to less than 4 Å (Fig. 2d). The number of amino acids involved in contacts in one CPN60β1 subunit is much less than that in one GroEL subunit, especially in the apical domain. Close inspection of inter-subunits in the apical domain of GroEL revealed that there are seven amino acids in one subunit and five amino acids in the adjacent subunit involved in contacts. On the other hand, there are only two amino acids in one CPN60 subunit and two amino acids in the adjacent subunit involved in contacts. All these amino acids are listed in the supplementary data (Additional file 1: Figure S2A). Weaker associations between subunits may explain the enhanced protease sensitivity of the CPN60β1 homo-oligomer relative to GroEL [39]. (3) Finally, CPN60β1 resembles the allosteric intermediate state. Superimposing single rings of CPN60β1 and apo GroEL revealed that the conformation of CPN60β1 resembled the intermediate state between apo GroEL (PDB: 1XCK) [43] and GroEL-ADP (PDB: 4KI8) [15] (Additional file 1: Figure S2B). Between α-carbon atoms of CPN60β1 and GroEL-Apo (1XCK), the root-mean-square deviation was computed as 2.1 Å, and 2.82 Å between α-carbon atoms of GroEL-ADP (4KI8) and GroEL-Apo (1XCK). Further evidence comes from the position of E209. In the CPN60β1 apo state, E209 is in an intermediate state and shifts less dramatically during allosteric movement (Fig. 2e), which could account for the influence of the apical domain on ATP turnover [17].

### An enlarged ATP binding pocket

**Fig. 3:**
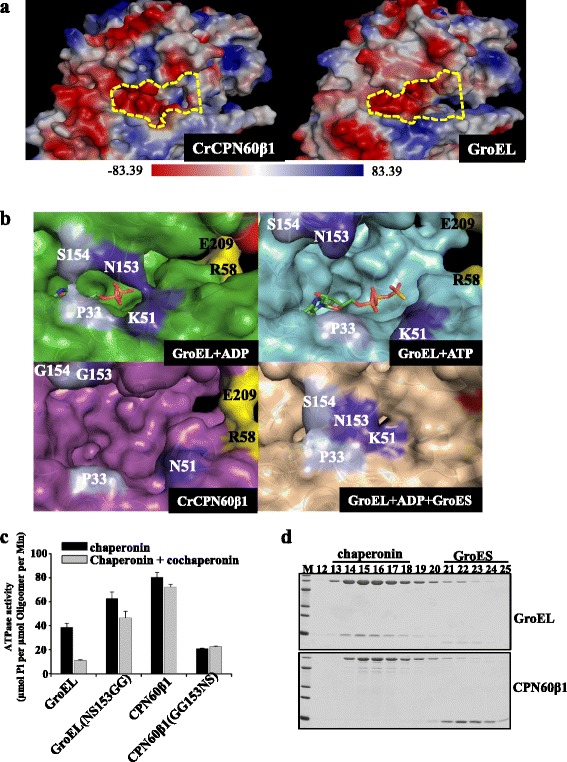
The ATP binding pocket in chaperonins. **a** Surface representation of ATP binding pocket in chaperonin oligomers. The ATP binding pockets are marked with yellow dashed lines and positive and negative amino acids in oligomers are colored in blue and red, respectively. **b** Comparison of ATP binding pockets in various conformations. N153 and K51 are colored in dark purple, and S154 and P33 are colored with light purple. **c** The inhibitory effect of co-chaperonin on ATPase activities of chaperonin and its mutant. The inhibitory effect of co-chaperonin GroES on GroEL and its mutant, or CrCPNs (a complex containing three proteins CrCPN23, CrCPN20, and CrCPN11 which are coexpressed in *E. coli*) on CPN60β1 and its mutant. The data was obtained with three independent experimental replicates and standard deviations are shown. **d** Interaction of chaperonin with GroES in the presence of AMP-PNP by gel filtration. A 50-μL reaction mixture containing 2 μM chaperonin and 10 μM GroES was separated on a Superdex 200 column with buffer containing 50 μM AMP-PNP. Collected fractions were resolved by SDS-PAGE and stained with Coomassie

We have reported that the ATPase activity of chloroplast chaperonin is 2- to 3-fold greater than that of GroEL and that co-chaperonin inhibits chloroplast chaperonin ATPase activity less substantially than GroEL. Both of these aspects may have evolved to optimally cope with the burden of folding extensive amounts of Rubisco [39]. Therefore, we curiously compared the ATP binding pocket of CPN60β1 and apo GroEL (Fig. 3a). The ATP binding pocket in CPN60β1 is wider, suggesting that adenosine may be more readily released. During GroEL allosteric progress, N153 and S154 in the intermediate domain close the adenosine binding pocket by interacting with K51 and P33 in the equatorial domain of GroEL-ADP or GroEL-GroES-ADP states, and the contacts are tighter in the GroEL-GroES-ADP conformation [11, 15, 45] (Fig. 3b). In CPN60β, positions 153 and 154 are occupied by glycine residues that have no side chains to aid closure of the ATP binding pocket upon co-chaperonin binding. To investigate the influence of side chains of these two amino acids during allosteric movement, we checked the co-chaperonin inhibitory effect on recombinantly purified double mutant chaperonin (Fig. 3c). GroES inhibited the ATPase activity of the double mutant GroEL (NS153GG) less significantly than GroEL (Fig. 3c). It is worth noting that the ATPase activity of GroEL (NS153GG) is higher than GroEL. Bioinformatic analysis revealed that glycine is conserved at position 153 in more than 100 Cpn60 proteins, suggesting that loose closure of the adenosine binding pocket may be a defining characteristic of Cpn60s that accounts for the weak inhibitory effect of co-chaperonins [39, 46]. When the two glycines in CPN60β1 were mutated to the corresponding amino acids of GroEL, the resulting mutant CPN60β1 (GG153NS) had much lower ATPase activity, which did not further decrease in the presence of co-chaperonin CrCPNs (Fig. 3c). In contrast to GroEL, the non-hydrolyzable ATP analogue AMP-PNP did not promote binding of GroES to CPN60β1 (Fig. 3d), and also did not induce CPN60β2 to move allosterically (Additional file 1: Figure S3), reinforcing that the ATP binding pocket in CPN60 is distinct.

### The equatorial domain directs the oligomer formation

**Fig. 4:**
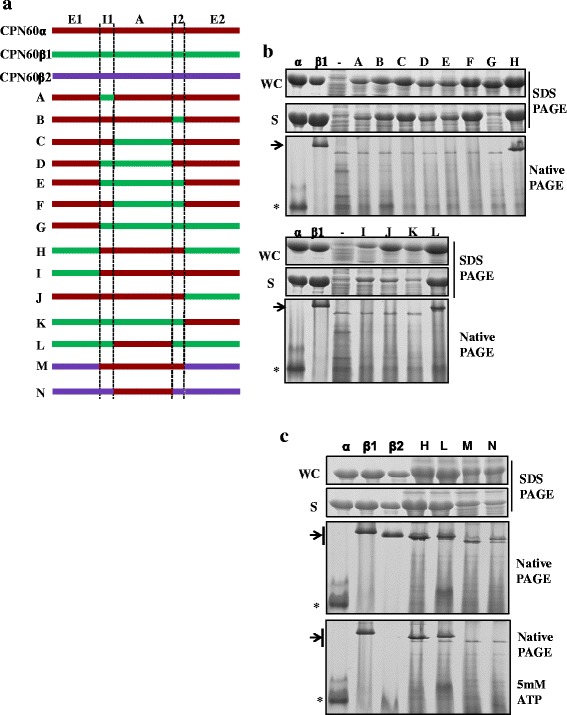
Segments influencing oligomer assembly and functionality. **a** Diagram of CPN60 subunits and the constructed chaperonin chimeras of CPN60s. Domain designation and amino acid numbering were used with respect to GroEL. E1 domain (1–137), I1 domain (138–190), A (191–374), I2 (375–409), E2 (410–548). **b** Solubility and oligomeric states of chaperonin chimeras. Whole cell lysate (WC) from 1 mL of induced cells were resolved with 12 % SDS-PAGE. The soluble fractions were separated with 12 % SDS PAGE and 6 % native PAGE. Purified CPN60α monomer, CPN60β1 oligomer, and uninduced cell lysate (–) were loaded as controls. Proteins were visualized by Coomassie staining. The positions of oligomers and monomers are indicated by an arrow and *, respectively. **c** Oligomer formation and disassembly of chaperonin chimeras. The whole cell lysates (WC) were analyzed by 12 % SDS-PAGE, and soluble fractions (S) were resolved with 12 % SDS-PAGE and 6 % native PAGE with or without incubation for 30 min at 25 °C with 5 mM ATP/Mg. Purified CPN60α monomer, CPN60β1 oligomer, and CPN60β2 oligomer were loaded as controls. The oligomer and monomer positions are indicated by an arrow and *, respectively. Proteins were visualized by Coomassie staining

CPN60β, but not CPN60α, forms oligomers [39]. To investigate the elements that influence chaperonin assembly, we constructed a series of chimeras with domains swapped between CPN60α and CPN60β1 (Fig. 4a). All chimeric proteins were induced to substantial amounts (Fig. 4b, WC panel), but the proteins tended to aggregate in chimeras with equatorial fragments originating from two different CPN60 subunits (Fig. 4a, b, chimeras G, I, J and K), indicating that certain interactions were disturbed. When the entire equatorial domain of CPN60β1 (E1 and E2) was present, the chimeras assembled into oligomers independent of the origin of the intermediate domains (chimeras H and L). The results were further confirmed by chimeras M and N, in which the CPN60β1 segments in H and L were changed to CPN60β2 segments (Fig. 4a, c). Consistent with the biochemical properties of individual homo-oligomers, i.e. CPN60β2 disassembles into monomer upon ATP hydrolysis while CPN60β1 does not [39], chimeras H, L, M, and N behaved like their non-chimeric counterparts containing the same equatorial domain (Fig. 4c). These results indicate that the equatorial domain not only directs oligomer formation but also responds to ATP hydrolysis.

### C-terminal (aa residues 484–547) of CPN60β influences oligomer disassembly driven by ATP

**Fig. 5:**
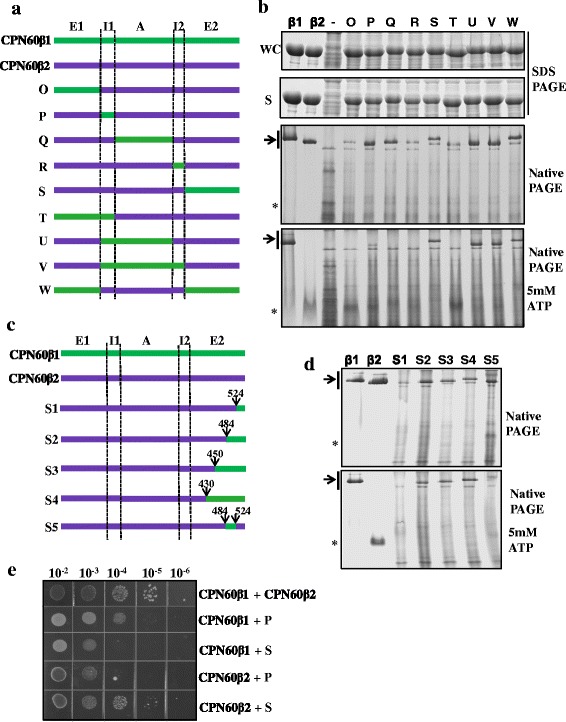
Segments influencing CPN60β homo-oligomer disassembly. **a** Diagram of chaperonin chimeras between CPN60β1 and CPN60β2. The domain designation and amino acid numbering are the same as described for Fig. 3a. **b** Oligomer formation and disassembly of chaperonin chimeras upon incubation with ATP. Visualization of the disassembly of CPN60β chimeric oligomers was performed as described in Fig. 3c. **c** Diagram of CPN60β subunits and the constructed chaperonin chimeras. The swapping positions are indicated in the figure. **d** Disassembly of chaperonin chimeras. The untreated soluble fractions and soluble fractions treated with 5 mM ATP/Mg were resolved by 6 % native PAGE and visualized by Coomassie staining. Recombinantly induced CPN60β1 and CPN60β2 oligomers were loaded as controls. The positions of oligomers and monomers are indicated by an arrow and *, respectively. **e** Functional replacement of GroEL by coexpression of CPN60β with chimeras. CPN60β chimeras and GroES were expressed in GroEL/ES-deficient *E. coli* strain MGM100. The strains were grown on medium supplemented with glucose and IPTG at 37 °C for 24 hours

CPN60β2 disassembles into monomers completely upon ATP hydrolysis [39]. ATP analogue (ADP-AlF_3_) promoted the disassembly of CPN60β2, but ADP and non-hydrolyzable ATP analogue (AMP-PNP) did not cause disassembly (Additional file 1: Figure S3). In the presence of ADP, but not AMP-PNP, the binding of co-chaperonin (CrCPN20) promoted CPN60β2 disassembly (Additional file 1: Figure S3). ATP binding and hydrolysis drive the allosteric movement of chaperonin oligomers, which rebuilds interactions between subunits [47]. Therefore, disassembly of CPN60β2 might be due to the failure to rebuild these interactions. To further assess how the elements in the equatorial domain respond to disassembly driven by ATP hydrolysis, we constructed chimeras with domain replacements between CPN60β1 and CPN60β2 (Fig. 5a). When the I1 domain of CPN60β2 was replaced by the same region of CPN60β1 (Fig. 5a, chimera P), CPN60β2 oligomers were stabilized to detectable levels upon ATP hydrolysis (Fig. 5b, bottom panel). In Fig. 5b, the bands under CPN60 oligomers were endogenous proteins of *E. coli* which also appeared in the negative control. Oligomer stability was enhanced when both I1 and A domains from CPN60β1 were inserted into the chimera (Fig. 5a, b, chimeras U and V), but oligomers were no longer stabilized when the both E1 and I1 domains from CPN60β1 were inserted instead (chimera T). These results indicate that the interaction between I1 and A domains stabilizes oligomers. We further analyzed these two domains in the crystal structure of CPN60β1 and GroEL, and found that G180 (amino acid numbered with respect to GroEL) may affect inter-subunit interaction between K181 (I1 domain) and E283 (A domain) (Additional file 1: Figure S4A). A series of CPN60β2 mutants (S180G, K181A, SK180GA) evidenced that serine 180 hindered the allosteric movement of the I1 domain of CPN60β2, preventing interaction with adjacent subunits (Additional file 1: Figure S4B).

More importantly, insertion of the E2 domain from CPN60β1 into chimeras substantially stabilized oligomers in the presence of ATP (Fig. 5a,b, chimeras S and W). We further divided the E2 domain into four segments according to sequence homologues and constructed segment swapping chimeras S1–S5 (Fig. 5c). Insertion of C-terminal fragments containing at least amino acid residues 484–547 of CPN60β1 into chimeras significantly stabilized oligomers upon ATP hydrolysis (Fig. 5c, d, chimeras S2, S3, and S4), whereas insertion of the most divergent fragment (aa residues 524–547, not visible in the crystal structure, chimera S1) or a relatively conserved fragment (aa residues 484–524, chimera S5) into the chimera had no effect on oligomer stability. The C-terminus (484–547) might stabilize CPN60β1 oligomeric states by providing large hydrophobic fragments. Replacement of amino acids in the CPN60β2 E2 domain with the corresponding amino acids of CPN60β1 yielded three further mutants (ET431RR, S451C, and RVMD468EVMK). CPN60β2 (ET431RR) and CPN60β2 (S451C) did not influence oligomer formation and disassembly upon ATP hydrolysis; however, CPN60β2 (ET431RR) changed the electrophoresis migration rate during native PAGE (Additional file 1: Figure S5A). CPN60β2 (RVMD468EVMK) did not assemble into oligomers, although a substantial portion of the protein is soluble, a phenomena similar to that observed with CPN60β2 (Q467N) (Additional file 1: Figure S5A and S5B). Structural analysis revealed that N467 and E468 of CPN60β1 form van der Waals forces between rings to stabilize the oligomers (Additional file 1: Figure S5C). Point mutants of some amino acids involved in inter-subunit interaction showed CPN60β2 (S46A) and CPN60β2 (S180G) stabilized oligomers to some extent upon ATP hydrolysis (Additional file 1: Figure S5B), and double mutants showed no cumulative effects (Additional file 1: Figure S5D). When these amino acids in CPN60β1 were replaced with the corresponding amino acids of CPN60β2, no obvious effect was observed (Additional file 1: Figure S5E). The inherent differences between the equatorial domains of CPN60β1 and CPN60β2 confer their differential response to ATP.

Individual CPN60 subunits could not complement GroEL function in *E. coli* [39]. Similarly, the chaperonin chimeras (H, L, M, N), with the equatorial domain from CPN60β subunits and the apical domain from the CPN60α subunit could not complement GroEL function (Additional file 1: Figure S6A,B). Coexpression of both CPN60β subunits could partially complement GroEL function after a long incubation time (Fig. 5e) [39]. *E. coli* MGM100 coexpressing CPN60β1 with chimera P, which contains the entire equatorial domain of CPN60β2, grew better than with chimera S, which has the E2 domain of CPN60β1 (Fig. 5e). Conversely, coexpression of CPN60β2 with chimera S complements GroEL function better than with chimera P (Fig. 5e), suggesting the equatorial domains from CPN60β subunits compensate for each other.

### CPN60α and CPN60β subunits are highly cooperative

**Fig. 6:**
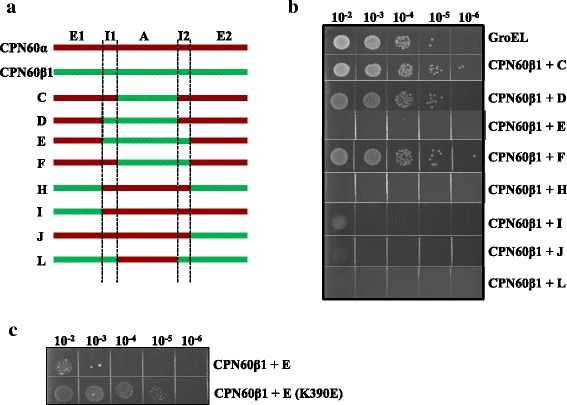
The cooperation of CPN60α and CPN60β. **a** Diagram of CPN60 subunits and the constructed chaperonin chimeras of CPN60s. The chimeras are same as Fig. 4a. **b** and **c** Functional complementation of GroEL by coexpression of CPN60β1 with chimeras. The experiments were performed the same as described in Fig. 1d, using GroES as co-chaperonin

CPN60 subunits could not complement GroEL function in *E. coli* alone, but the combination of CPN60α and CPN60β was functional [39]. To investigate the cooperation of both subunit types, we determined whether the constructed chimeras (Fig. 6a) could cooperate with CPN60β1 to complement GroEL function upon coexpression in GroEL-deficient *E. coli* strain MGM100 [48]. As shown in Fig. 6b, the intact equatorial domain and at least one part of the intermediate domain from CPN60α is indispensable for functional cooperation with CPN60β1 (CPN60β1 + C, D, F). The presence of one segment of the equatorial domain or the entire intermediate domain from CPN60β1 abolished the functional cooperation required for growth (CPN60β1 + E, H, I, J, L). These results indicated that the equatorial domain of CPN60α could not support subunit assembly, but cooperated with CPN60β1 to form functional oligomers that complement GroEL function. We further mutated chimera E (K390E, located in I2 domain) to disturb the salt bridge formed with E179 (located in I1 domain) in the CPN60β1 subunit. The co-expression of chimera E (K390E) and CPN60β1 could partially complement GroEL function (Fig. 6c), suggesting that strong interaction between the two segments of the intermediate domain in CPN60β1 hinder the allosteric movement of chaperonin.

**Fig. 7:**
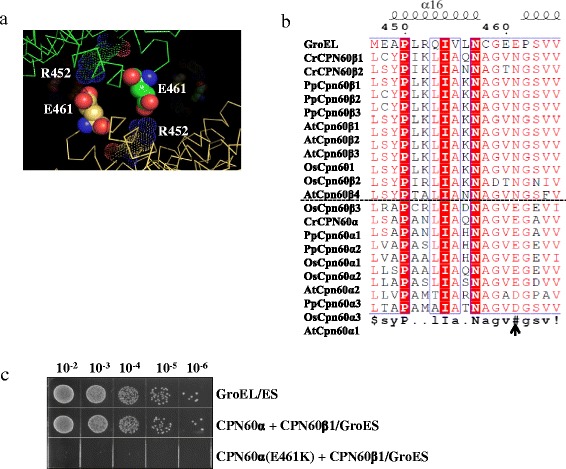
The inter-ring salt bridge between R452 and E461. **a** A view of inter-ring salt bridge between R452 and E461 in GroEL. **b** Structural alignment of Cpn60α and Cpn60β subunits from four organisms. The four organisms are *Chlamydomonas reinhardtii* (Cr), *Oryza sativa* (Os), *Physcomitrella patens* (Pp), and *Arabidopsis thaliana* (At). Cpn60 sequences are aligned with MultAlin and Espript. Position 461 was indicated by *arrows*. **c** Functional complementation of GroEL by coexpression of CPN60β1 with CPN60α/mutant. The experiments were performed as described in Fig. 1d. The coexpression of CPN60β1, CPN60α/mutant, and GroES was induced with 1 mM IPTG in GroEL-deficient *E. coli* strain MGM100. The strains were grown on medium supplemented with glucose and IPTG at 37 °C for 15 hours

In GroEL, communication between its two rings is mediated by a close salt bridge formed by R452 and E461 [49, 50] (Fig. 7a). Sequence alignment revealed that N461 is conserved in more than 50 Cpn60β subunits, whereas a negatively-charged amino acid (E461 or D461) is conserved at the same position in Cpn60α subunits. On the other hand, a positively-charged amino acid (K452 or R452) is conserved at position 452 in Cpn60β subunits, while neutral amino acids are prevalent at this position in Cpn60α subunits, except for CPN60α from *Chlamydomonas* which has R at position 452 (Fig. 7b). To test whether E461 connected two rings as that of GroEL, the mutant CPN60α (E461K) was coexpression with CPN60β1 and GroES to complement GroEL function (Fig. 7c). Once this residue was replaced by lysine, CPN60 could not rescue *E. coli* growth from the GroEL deletion. Thus, E461 of CPN60α is essential for the functionality of CPN60 oligomers.

## Discussion

The multi-subunit chloroplast chaperonin Cpn60 is more complex in composition relative to its prokaryotic homologue GroEL. Cpn60, consisting of both Cpn60α and Cpn60β types, is functional both in vivo and in vitro [6, 33, 51], whereas the homo-oligomeric Cpn60β type is only functional with selective co-chaperonins in vitro [36, 52]. Recombinantly purified *Chlamydomonas* CPN60β1 homo-oligomers exhibit biochemical properties similar to functional hetero-oligomers, e.g. possessing high ATPase activity and high protease sensitivity, being binding model substrates and interacting with co-chaperonins [39]. However, CPN60β1 homo-oligomers were not fully functional in refolding model substrates [39] (Fig. 1), which might result from a failure of allosteric movement during the functional cycle (Fig. 5). Nonetheless, due to the described functional similarity, the structure of CPN60β1 probably resembles the structure of chaperonin hetero-oligomers in vivo and is not a result of a crystallization artefact of a non-functional and, therefore, irrelevant protein. Indeed, the loosely-compacted crystal structure of CPN60β1 was consistent with the observation that Cpn60 oligomers are highly susceptible to proteolytic degradation. It is not clear why the chloroplast chaperonin might adopt a loosely compacted structure. Considering the plastid-specific co-chaperonin Cpn20 that consists of a double GroES-like domain, it is plausible that a loosely-compacted structure has been optimized to interact with Cpn20. Admittedly, the presented CPN60β1 structure may also deviate significantly from the active Cpn60 structure in vivo; however, it represents a first step toward structural and functional understanding of chloroplast chaperonin and will likely provide insights that can be exploited by future structural studies.

### An enlarged nano-cage for substrate folding in CPN60

The folding nano-cage present in the crystal structure of GroEL/GroES is about 175,000 Å^3^ and could theoretically accommodate substrate proteins of up to 70 kDa [11]. In reality, the functional volume of this nano-cage is smaller, probably because the 23 C-terminal amino acids not resolved in the GroEL/GroES structure protrude into the cage and occupy space. Most GroEL substrates are actually smaller than 50 kDa [53]. In the crystal structure of CPN60, the diameter of the central cavity is about 6 Å longer than the diameter of the GroEL cage, but the largest Cpn60 substrate identified to date is the Rubisco large subunit, of only around 50 kDa in size [33, 54, 55]. Tang et al. [56] reported that a GroEL cage volume increased by 2–5 % remarkably decelerated the folding of large substrates, e.g. MBP (41 kDa) and RrRubisco (50 kDa), by more than 2-fold. Consistent with their results, we found that the increased size of the Cpn60 cavity corresponded to a slower rate of folding of RrRubisco relative to GroEL [46]. Chaperonin folding of endogenous CrRubisco was not investigated in this study due to the inherent difficulty associated with the biogenesis of green type Rubisco in *E. coli*.

### The decisive role of Cpn60α is supported by its unique equatorial domain

It is reported that the Cpn60α deletion is lethal to plants [57–59]. Here, we found that CPN60α is highly cooperative with CPN60β1 to form functional oligomers potentially by regulating the allosteric movement of CPN60 oligomers (Figs. 6 and 7). Replacement of the CPN60α apical domain by the same domain of CPN60β1 did not influence subunit cooperation and oligomer functionality, suggesting that the apical domain of CPN60α does not play an essential role (Fig. 6b). The equatorial domain of Cpn60β type, but not Cpn60α, directs the subunits to form oligomers, suggesting that the Cpn60β subunits initiate oligomer assembly [39] (Fig. 4). Homo-oligomeric Cpn60β type possesses high ATPase activity, substrate binding ability and interaction with co-chaperonins, and it is partially functional with selective co-chaperonin. The equatorial domain and at least one intermediate segment from CPN60α are indispensable to the functionality of oligomers (Fig. 4), suggesting that fragments from Cpn60α offer Cpn60β subunit flexibility during its allosteric movement. Unlike its GroEL homologue, Cpn60 exhibits sophisticated regulation between its subunits.

## Conclusions

In our study, the first crystal structure of *Chlamydomonas* chloroplast chaperonin homo-oligomer (CPN60β1) was solved at 3.8 Å, and displayed a rigid body and structural features such as looser compaction, a larger central cavity, less contact sites, and an enlarged ATP binding pocket compared to GroEL. Interestingly, we found that the overall structure of Cpn60 resembles the GroEL allosteric intermediate state. There are two amino acids (153 and 154) conserved among Cpn60s which were involved in regulation of ATPase activity by co-chaperonins. Our domain swapping analysis revealed that the equatorial domain of CPN60α maintains its monomeric state in the absence of CPN60β (CPN60β1 or CPN60β2). This equatorial domain of CPN60α was also indispensable for functional cooperation with CPN60β1. We additionally discovered that the C-terminal fragment (aa residues 484–547) of CPN60β type influenced oligomer disassembly and allosteric rearrangement driven by ATP hydrolysis. More interestingly, two CPN60β subunits compensated for each other to rescue *E. coli* growth partially. Our results shed light on chloroplast chaperonin structure and cooperation between CPN60 subunits.

## Methods

### Plasmids cloning and protein purifications

Plasmid cloning for expression of the mitochondrial Hsp10, chaperonin chimeras, and chaperonin mutants, as well as purification of proteins are described in Additional file 1: Material and Methods.

### Crystallizations and X-ray data collection and processing

Crystallizations of CPN60β1 oligomers and X-ray data collection and processing are described in Additional file 1: Material and methods.

### *E. coli* complementation

Analysis to determine whether chaperonin chimeras complement GroEL function was performed as described previously [39]. All chimeric or point-mutated forms of chaperonin were constructed into pOFX vector [60], and the resulting plasmids were transformed into *E. coli* MGM100 cells by electroporation (MicroPulser™, Bio-Rad). A single transformant colony was picked and grown in LB medium supplemented with 30 μg/mL kanamycin, 20 μg/mL spectinomycin and 0.02 % arabinose to an OD_600_ of approximately 1; 1 mL of cells was collected, carefully washed five times and resuspended in 1 mL LB. Ten-fold dilution series were made, and 7.5 μL of diluted cells were spotted onto LB agar plates supplemented with 0.2 % glucose/1 mM IPTG, 30 μg/mL kanamycin and 20 μg/mL spectinomycin. The cells were grown at 37 °C for 12–13 hours.

### ATPase activity

The ATPase activity of chaperonin was measured using a coupled enzymatic assay as described previously [61]. ATP hydrolysis by chaperonin is linked to the oxidation of NADH to NAD+ by the coupled reaction of Pyruvate kinase and L-lactate dehydrogenase. After sequential addition of 1 mM phosphoenolpyruvate, 20 U/mL pyruvate kinase, 30 U/mL lactate dehydrogenase, and 0.5 mM NADH into the reaction buffer (50 mM HEPES/KOH pH 7.5, 10 mM KCl, 10 mM MgCl_2_), 1 mM ATP and/or 0.5 μM co-chaperonin was added and incubated for 3 min at 25 °C. The absorbance at 340 nm was monitored immediately after addition of 0.2 μM chaperonin for 10 min.

### Interaction assay

Analysis of chaperonin chimera and co-chaperonin interaction was performed with analytical gel filtration as described previously [61]. Briefly, 2 μM CPN60β1 chaperonin and 10 μM co-chaperonin were incubated in buffer containing 20 mM MOPS-KOH, pH 7.5, 5 mM Mg(OAc)_2_, 100 mM KCl, and 2 mM ADP-AlF_3_ for 30 minutes at 25 °C, and the protein complexes were then separated with a Superdex 200 PC3.2/10 column (GE Healthcare) with the same buffer but using only 50 μM ADP-AlF_3_ at a flow rate 0.05 mL/min. Finally, 60-μL fractions were collected, resolved by 15 % SDS-PAGE and visualized with Coomassie staining.

### Transmission electron microscopy

ATP (2 mM) was added to the incubation buffer (30 mM KF, 20 mM Tris-HCl pH 7.5, 10 mM MgCl_2,_ 3 mM KAl(SO_4_)_2_). The final concentrations of chaperonin (CPN60β1) and co-chaperonin (GroES) were 0.1 μM and 0.5 μM, respectively. The reaction was incubated at 4 °C for 30 min, and the solution was subjected to ultrafiltration to remove chemical precipitates. Then, 4 μL of the supernatant was placed onto the carbon-coated grids, which had been previously hydrophilized by glow discharge; 1 min later, excess protein sample was removed by filter paper. The specimen was stained with 1 % uranyl acetate for 1 min and allowed to air dry. Images were recorded by a CCD camera system (Olympus, Tokyo, Japan) at an accelerating voltage of 120 keV on a transmission electron microscope (FEI, Eindhoven, The Netherlands).

### Protein refolding and Rubisco carboxylation assay

Refolding of model substrates RrRubisco and Rubisco carboxylation assay were carried out as described previously [46, 62].

## Availability of data and materials

Coordinates and structure factor amplitudes for CPN60β1 oligomers are deposited in the Protein Data Bank under accession codes 5CDI.

Full Methods and any associated references are available in the Additional files.

## Additional file

Additional file 1: Supplementary data including six figures, one table and more experimental procedures can be found enclosed with this article. **Figure S1.** Refolding RrRubisco by chaperonin oligomers. **Figure S2.** Comparison of equatorial domains from CPN60β1 and GroEL. **Figure S3.** Disassembly of CPN60β2 in the presence of co-chaperonins or nucleotides. **Figure S4.** Structural features of CPN60β oligomers. **Figure S5.** Disassembly of chaperonin mutants. **Figure S6.** Functionality of chaperonin chimeras. **Table S1.** Data collection and refinement statistics. (PDF 1271 kb)
