# Supplementary material for: Structural insight into the cooperation of chloroplast chaperonin subunits
Source: BMC Biol. 2016 Apr 12;14:29. doi: 10.1186/s12915-016-0251-8 (PMC4828840; doi:10.1186/s12915-016-0251-8)
Supplement: Additional file 1: — Supplementary data including six figures, one table and more experimental procedures can be found enclosed with this article. Figure S1. Refolding RrRubisco by chaperonin oligomers. Figure S2. Comparison of equatorial domains from CPN60β1 and GroEL. Figure S3. Disassembly of CPN60β2 in the presence of co-chaperonins or nucleotides. Figure S4. Structural features of CPN60β oligomers. Figure S5. Disassembly of chaperonin mutants. Figure S6. Functionality of chaperonin chimeras. Table S1. Data collection and refinement statistics. (PDF 1271 kb) [file 12915_2016_251_MOESM1_ESM.pdf]

Supplementary methods

Plasmids cloning

Mitochondrial Hsp10 was amplified by PCR from mouse cDNA (a kind gift from Prof. Zhiheng Xu), and the resulting fragments were cloned between *NdeI/BamHI* restriction sites of pET22b vector to generate *Hsp10-pET22b.* The chaperonin chimeras with swapped domains were constructed by the seamless clone kit (Genebankbios). The point mutants were produced with a site directed mutagenesis method (Stratagene). All plasmids were verified by DNA sequencing.

Protein purification

CrCPN60β1 was purified as described previously [1]. Hsp10 was purified using the following procedure. Briefly, 1 liter *E. coli* BL21 (DE3) Gold cells containing *Hsp10-pET22b* were grown to an OD600 of about 0.6, induced by addition of 0.5 mM IPTG for 15 hours at 16°C and harvested by centrifugation. The cells were then resuspended in lysis buffer (50 mM NaH_2PO_4, pH 8.0, 300 mM NaCl, 10 mM imidazole and 1 X complete protease inhibitor cocktail (Roche)). Cells were broken by sonication, the debris was removed by centrifugation at 20,000 g for 30 minutes, and the resulting supernatant was loaded onto a 3 ml Ni–NTA agarose column (Merck) that had been equilibrated with lysis buffer containing 10 mM imidazole. The column was washed sequentially with lysis buffer containing 10 mM and then 25 mM imidazole and the protein was eluted with lysis buffer containing 250 mM imidazole. The collected protein was concentrated and quantified.

Crystallizations

CPN60β1 oligomers were screened for crystallization by using the sitting drop vapor diffusion method at 4 and 18°C (drop: 2μl; reservoir solution: 100 μl). The first condition in which leaf-shaped crystals were observed was in 8% v/v PEG 6,000, 0.15M NaCl, and 0.1M Tris-HCl pH 8.0 at 18°C after 8 hours. After hanging drop vapor diffusion was

optimized, we used MicroMax 007HFM IP to test large cuboid crystals, but their diffraction capability was very poor. The second condition (10% w/v Polyethylene glycol monomethyl ether 5,000, 5% v/v Tacsimate pH 7.0, and 0.1 M HEPES pH 7.0) formed cube-shaped crystals of very small size (0.05mm×0.05mm×0.05mm). This crystal was also optimized by the hanging drop vapor diffusion method at 18°C (drop: 1.2 µl 8 mg/ml protein and 1.5 µl crystallization reagent; reservoir solution: 500 µl). Cube-shaped crystals were observed after 2-3 days, but the crystals diffracted weakly again, only 6 Å. We also tried micro- and macroseeding, crystal dehydration and annealing, and even mutated the surface residues, lysine to alanine, and truncated the C-terminal, but the resolution did not improve. At last, the sandwich method was tried [2], in which the drop is between mica and silanization glass. The resulting crystals grown one week were briefly soaked in reservoir solution with the addition of 25% (v/v) glycerol, then flash-frozen in liquid nitrogen. For these crystals, the resolution reached 3.8 Å.

X-ray Data Collection and Processing

The X-ray diffraction data were collected at Shanghai Synchrotron Radiation Facility (SSRF) beamline 17U, China. Data was processed with HKL2000 [3]. The structures was solved by molecular replacement (MR) in PHENIX. For CPN60β1 oligomers, we used GroEL-ATP (PDB:1XCK) as the search model. The electro density did not fit well in the apical domain initially. After the structure of CPN60β1 apical-domain had been solved, several cycles of model building were done manually for the same domain in oligomers by COOT, then PHENIX was used to do the rigid refinement. Further rounds of refinement were carried out with the reference model apo GroEL(PDB:1XCK) using the phenix.refine program implemented in the PHENIX package [4] with energy minimization, group and individual B factor refinement, and TLS refinement. Molecular replacement and structure refinement were completed by COOT and PHENIX.

Supplementary figure legends

Figure S1. Refolding RrRubisco by chaperonin oligomers.

50 μM chemically denatured Rubisco was diluted 100-fold into buffer containing 0.5 μM GroEL or CPN60β1 and 1 μM co-chaperonins. 2 mM ATP was added to initiate the refolding reaction, and reactions were incubated for 1 h at 25°C, then stopped with 10 mM glucose and 2.5 U hexokinase. Rubisco carboxylation activity was measured from 30 μl samples after refolding.

Figure S2. Comparison of equatorial domains from CPN60β1 and GroEL.

(A) Contacts between adjacent subunits. The two adjacent subunits in the upper ring were colored in green and cyan, and the contacted subunit in the lower ring was colored in pink. The contact regions in the apical domain between two adjacent subunits are enlarged in the upper picture and the amino acids involved in contacts were listed in the bottom table.

(B) Structural comparison of CPN60β1 and GroEL-ADP with apo GroEL in their single ring forms. The apo GroEL(PDB:1XCK) is colored in grey. CPN60β1 and GroEL-ADP (PDB:4KI8) are colored pink and yellow respectively.

Figure S3. Disassembly of CPN60β2 in the presence of cochaperonins or nucleotides. Analytical gel filtration of CPN60β2-cochaperonin complexes. A 50 μl reaction mixture containing 2 μM CPN60β2, with or without 10 μM cochaperonins and 2 mM nucleotides as indicated was run on a Superdex 200 column. Collected fractions were resolved by SDS-PAGE and stained with Coomassie.

Figure S4. Structural features of CPN60β oligomers.

(A) Structural representation of K181 from one subunit and E283 from the adjacent subunit. The two subunits are colored in green and pink.

(B) Disassembly of CPN60β2 mutant oligomers upon ATP incubation. Soluble fractions with or without 5 mM ATP/Mg were resolved using 6% native PAGE and visualized

by Coomassie staining. Purified CPN60β1 and CPN60β2 oligomers were loaded as control. The positions of oligomers and monomers are indicated by arrow and * respectively.

Figure S5. Disassembly of chaperonin mutants.

(A) Disassembly of CPN60β2 mutants upon ATP incubation. Soluble fractions were resolved using 12% SDS PAGE and 6% native PAGE with or without 5 mM ATP/Mg, and visualized by Coomassie staining. Purified CPN60β1 and CPN60β2 oligomers were loaded as control. The positions of oligomers and monomers are indicated by arrow and * respectively.

(B) Disassembly of single amino acid mutants of CPN60β2. Some amino acids of CPN60β2 that are potentially involved in inter-subunit interaction were mutated into the corresponding amino acid of CPN60β1. The disassembly properties were analyzed as in Figure S6A except that the loading control was uninduced cell lysate.

(C) Structural representation of inter-ring interaction formed by N467 and E468. The two subunits in the upper ring and lower ring are colored in green and pink respectively.

(D) Disassembly of double amino acid mutants of CPN60β2 (S46A, S180G). Soluble fractions with or without 5 mM ATP/Mg were resolved using 6% native PAGE and visualized by Coomassie staining. Oligomers are indicated by an arrow.

(E) Oligomeric states of CPN60β1 mutants. The same positions shown in Figure. S6B were mutated in CPN60β1 into the corresponding amino acid of CPN60β2. The disassembly properties were analyzed as Figure S6B.

Figure S6. Functionality of chaperonin chimeras.

(A) Diagram of CPN60 subunits and constructed chaperonin chimeras of CPN60s. Domain designation and amino acids numbering were used based on GroEL. E1 domain (1-137), I1 domain (138-190), A (191-374), I2 (375-409), E2 (410-548).

(B) Chaperonin chimeras complement GroEL function. Chaperonin chimeras were

induced with 1 mM IPTG in *E . coli* strain MGM100, in which the expression of endogenous GroEL/ES is inhibited by the presence of glucose. The strains were grown on medium supplemented with glucose and IPTG at 37°C for 13 hours.

Supplementary references

1. Bai C, Guo P, Zhao Q, Lv Z, Zhang S, Gao F, Gao L, Wang Y, Tian Z, Wang J *et al*: **Protomer Roles in Chloroplast Chaperonin Assembly and Function**. *Mol Plant* 2015, **8**(10):1478-1492.
2. Sun L, Li J, Xu C, Yu F, Zhou H, Tang L, He J: **The sandwich method for protein crystallization and its effect on crystal growth**. *Acta Biochim Biophys Sin (Shanghai)* 2010, **42**(5):332-336.
3. Otwinowski C, Minor W: **Processing of x-ray diffraction data collected in oscillation mode**. *Methods Enzymol* 1997, **276**:307–326.
4. Adams PD, Afonine PV, Bunkoczi G, Chen VB, Davis IW, Echols N, Headd JJ, Hung LW, Kapral GJ, Grosse-Kunstleve RW *et al*: **PHENIX: a comprehensive Python-based system for macromolecular structure solution**. *Acta Crystallogr D Biol Crystallogr* 2010, **66**(Pt 2):213-221.

Figure S1 Zhang et al.

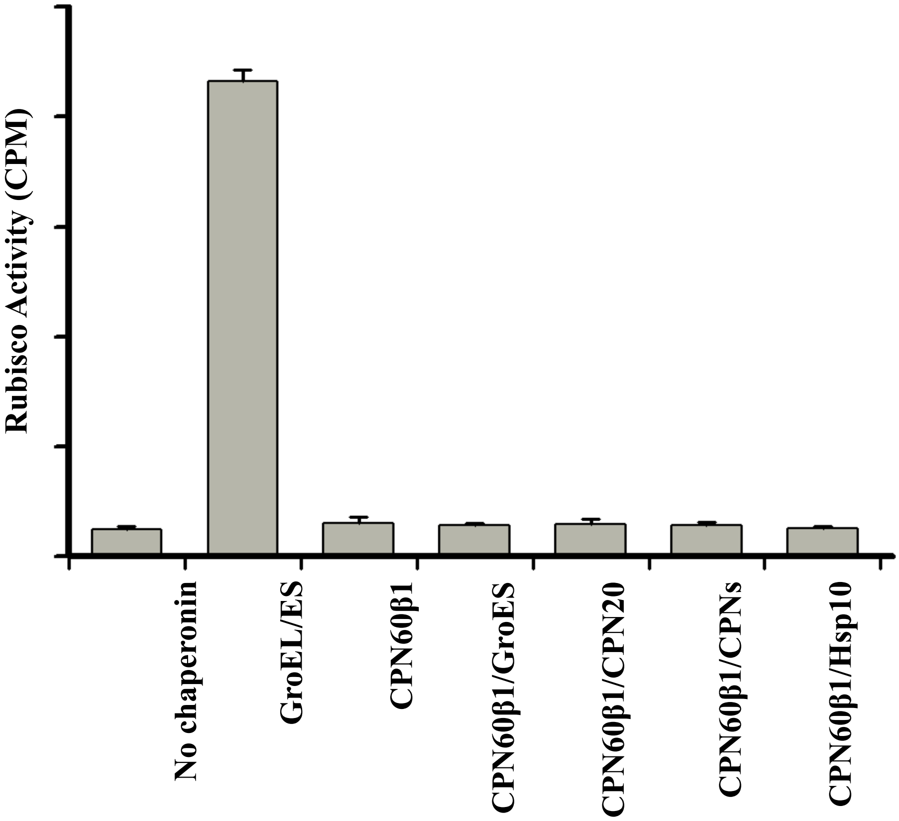

Figure S2 Zhang et al.

A

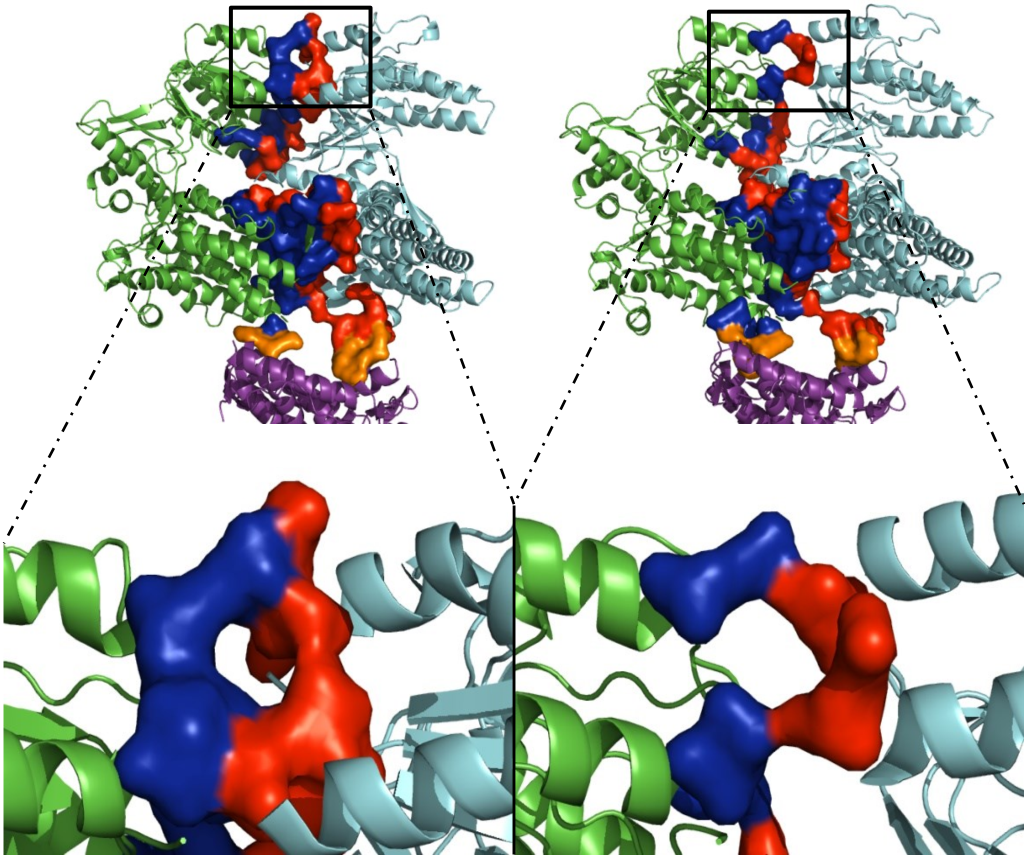

GroEL CPN60β1

GroEL A chain	GroEL G chain	CPN60β1 A chain	CPN60β1 G chain
5	7	2	2
226,228,229,231,257	216,241,242,243,269,271,272	232,258	245,269

B

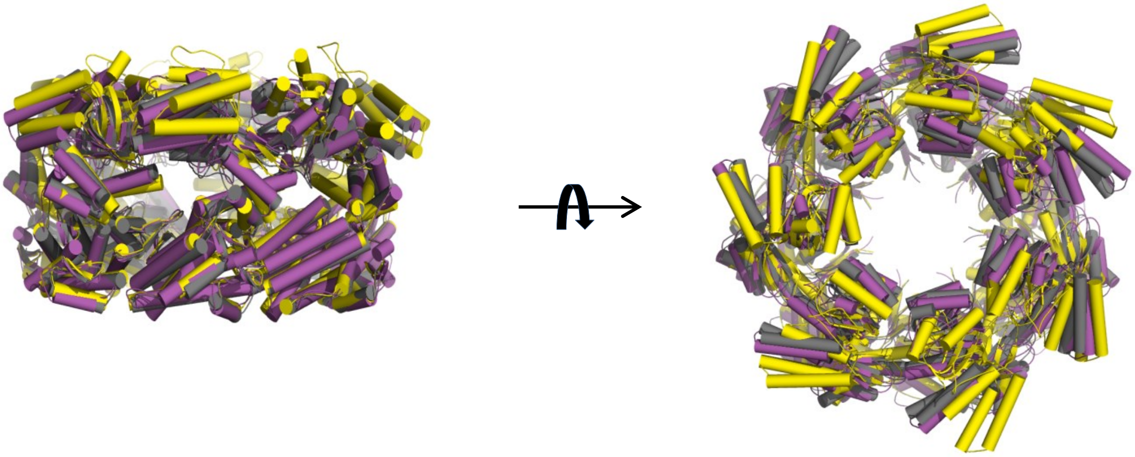

Figure S3 Zhang et al.

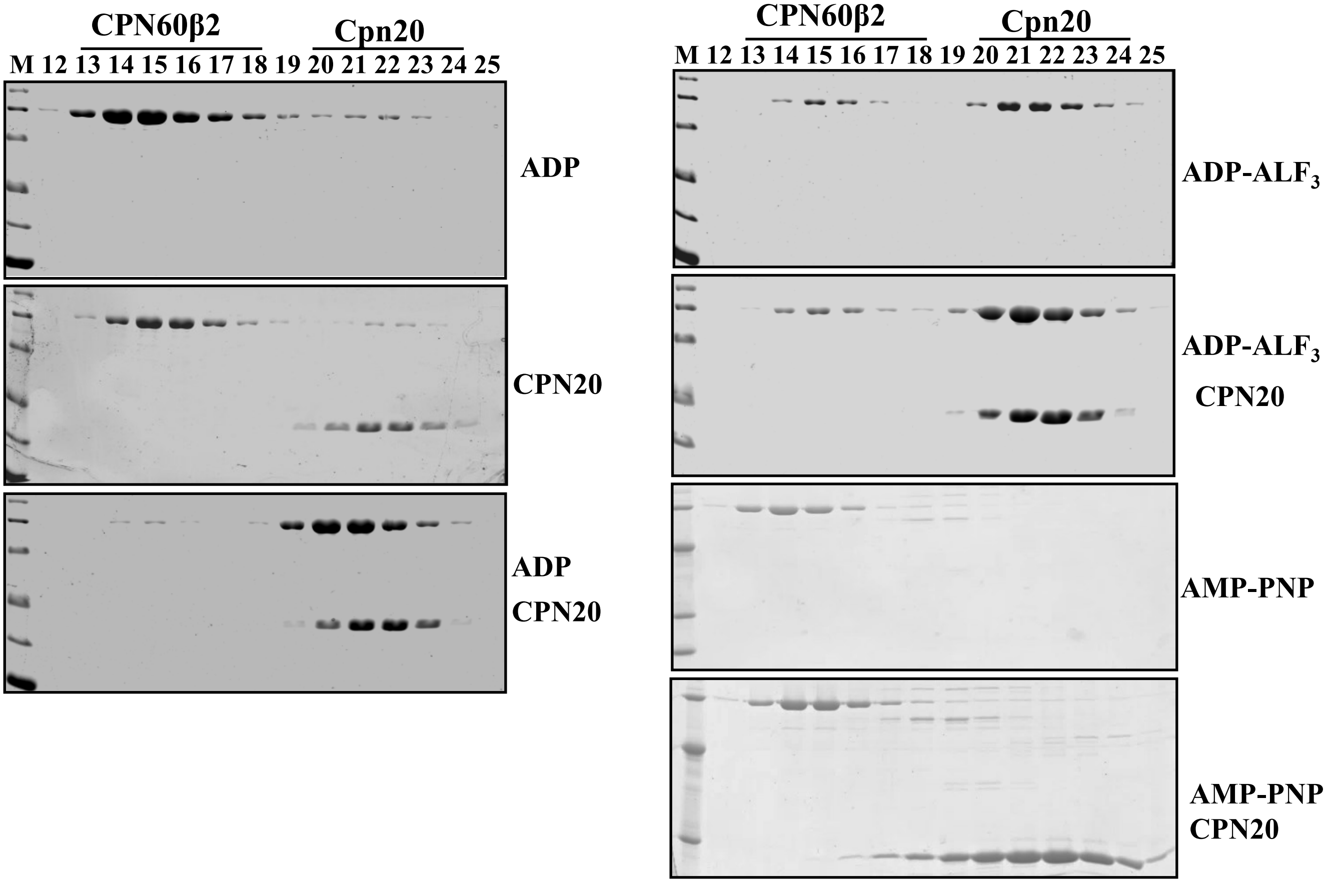

Figure S4 Zhang et al.

A

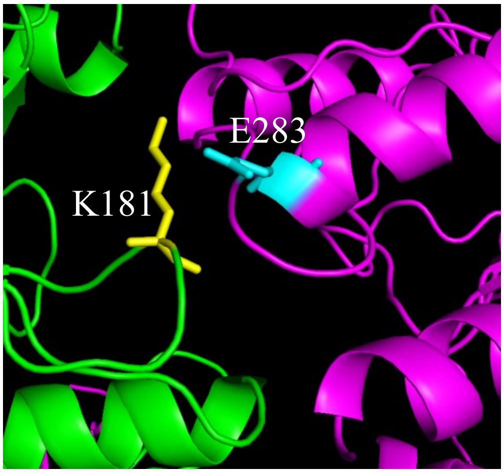

B

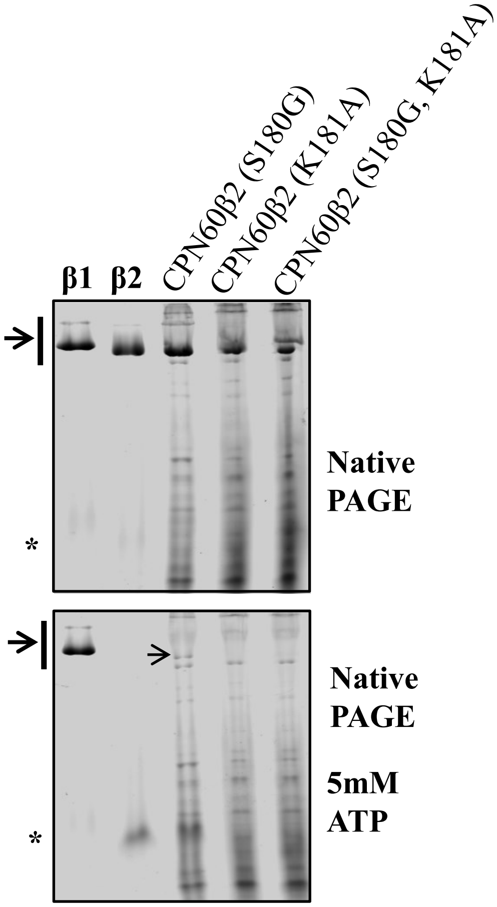

Figure S5 Zhang et al.

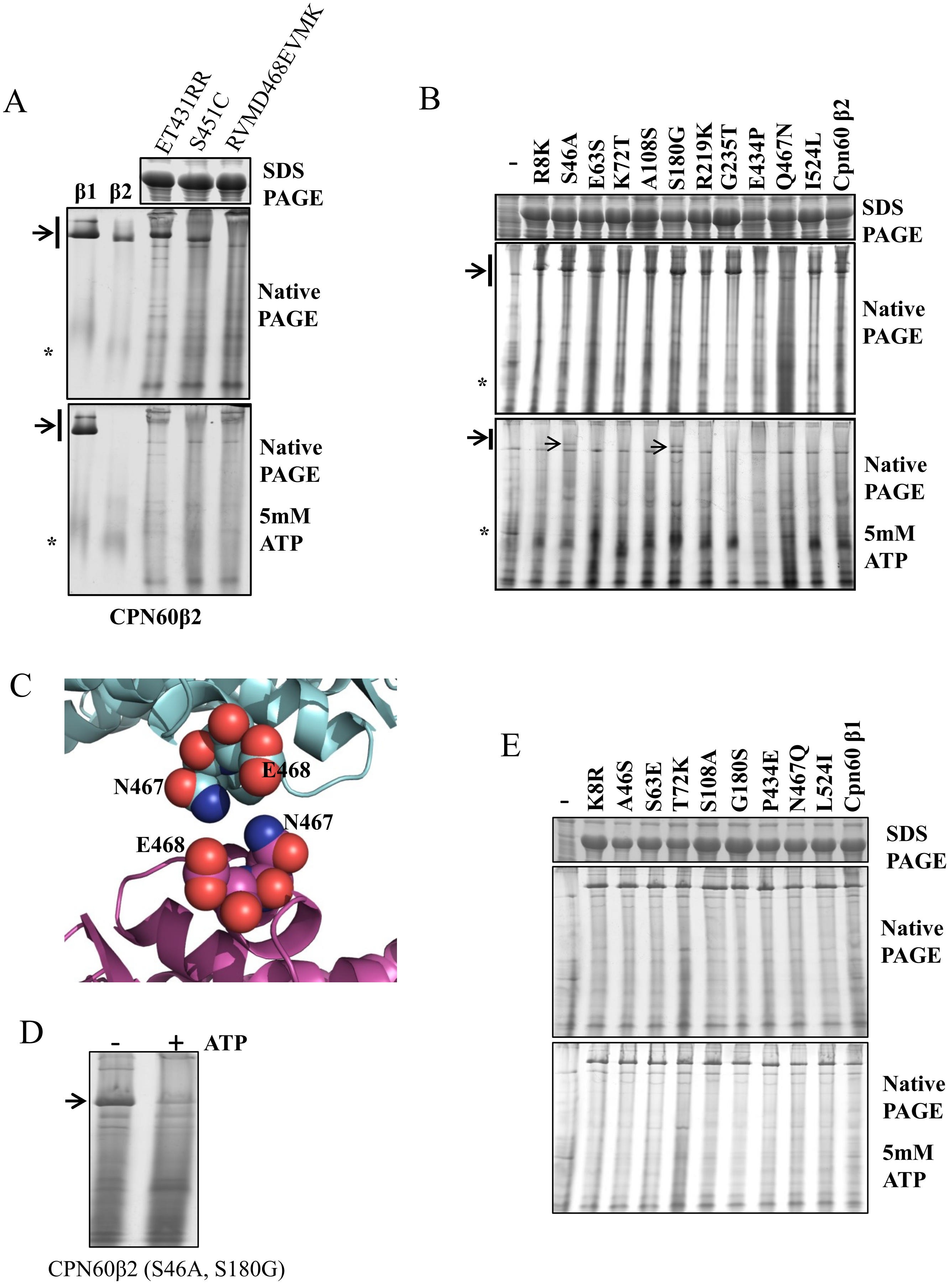

Figure S6 Zhang et al.

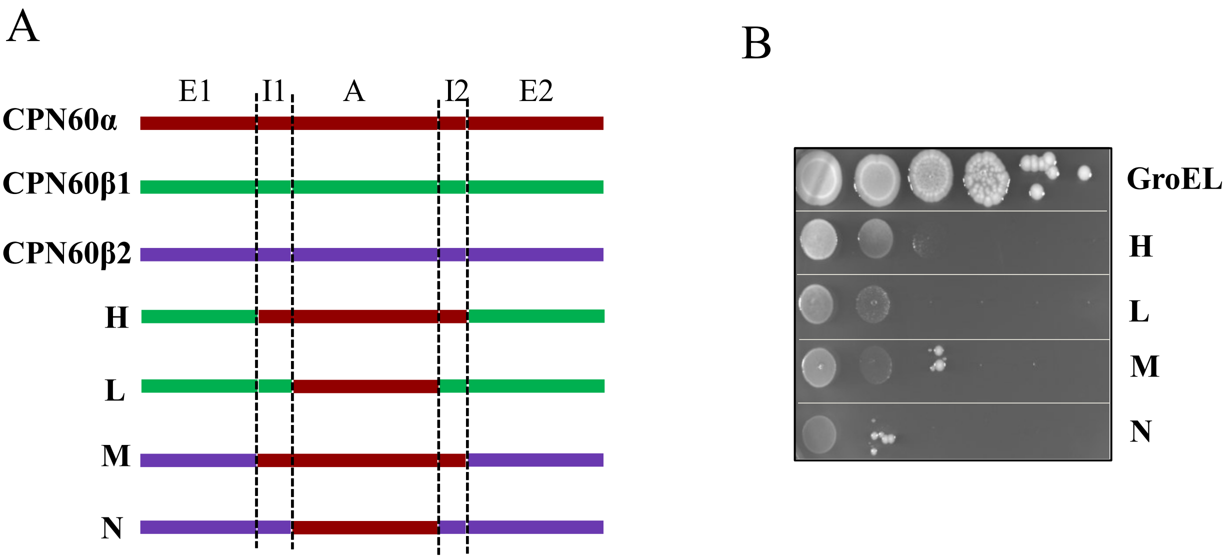

Table S1. Data collection and refinement statistics

	CPN60β1
**Space group**	P 1 21 1
**Cell dimensions**	
**a, b, c (Å)**	143.34 174.39 213.68
**α, β, γ (°)**	90.00 94.69 90.00
**Resolution (Å)**	48.66-3.8 (3.87-3.8)
**R merge**	0.104 (0.97)
**I / σI**	18.27 (1.43)
**Redundancy**	4.0 (3.8)
**Completeness (%)**	96.7 (93.6)
**Refinement**	
**R_{work}**	0.275
**R_{free}**	0.325
**Average B factors**	187.3
**r.m.s. deviations**	
**Bond length (Å)**	0.002
**Bond angles (°)**	0.6
**No. Waters**	-
